# Narratives about Breaking the Cycle of Intergenerational Violence in Families: Different Routes, Same Goal

**DOI:** 10.1007/s40653-026-00961-9

**Published:** 2026-08-11

**Authors:** Laura Mielityinen, Taina Laajasalo, Eija Paavilainen

**Affiliations:** 1 Tampere University, Tampere, Finland; 2 Finnish Institute for Health and Welfare, Helsinki, Finland

**Keywords:** Intimate partner violence, Child maltreatment, Intergenerational transmission of violence, Narrative analysis

## Abstract

Intergenerational violence in families is well documented, yet research often relies on quantitative methods, overlooking the complexity of lived experiences. Qualitative studies on parents exposed to both intimate partner violence (IPV) and child maltreatment remain scarce. This study examines narratives of Finnish parents to explore how parents with childhood experiences of both maltreatment and exposure to IPV narrate and make meaning of intergenerational continuity or change in violence. Data consist of 15 written narratives analyzed through narrative methods, including composite narratives and fictionalized characters to synthesize accounts and highlight thematic patterns. Our findings disrupt the notion of a simple, linear cycle of harm. Instead, intergenerational transmission emerges as a dynamic and probabilistic process. Breaking one cycle, such as IPV, does not automatically end another, like child maltreatment. What seems to matter is how individuals make sense of and integrate relational traumas into their life story. When these experiences are acknowledged and processed, parents can move toward earned secure attachment, transforming both parenting and partner relationships. Together these insights highlight the need for prevention strategies that go beyond exposure, focusing on early attachment support and interventions that combine personal agency and re-authoring childhood experiences with external resources such as safe relationships and professional guidance.

**Supplementary Information:**

The online version contains supplementary material available at https://doi.org/10.1007/s40653-026-00961-9.

## Introduction

Research consistently highlights the co-occurrence of intimate partner violence (IPV) and child maltreatment within the same family unit (Herbert et al., 2023; Mielityinen et al., 2024). Research also suggests that individuals exposed to violence in childhood—either as victims of maltreatment or as witnesses to IPV—are at heightened risk of perpetuating these patterns in adulthood, including perpetrating IPV or child maltreatment or experiencing IPV in their own intimate relationships (Assink et al., 2018; Curtis et al., 2023). Furthermore, studies indicate that dual exposure to both maltreatment and IPV during childhood further amplifies this risk, making these individuals significantly more likely to either perpetrate or experience violence later in life than those exposed to only one form of violence (Herbert et al., 2023; Hultmann et al., 2020; Shields et al., 2020).

Existing studies offer important quantitative evidence regarding the prevalence of IPV and child maltreatment co-occurring within the same family system and the likelihood of one of these individual forms of violence persisting and being intergenerationally transmitted (Madigan et al., 2019). Moreover, they identify critical risk (e.g. maternal anxiety) and protective factors (e.g. social support) that influence these trajectories, as well as adverse outcomes associated with co-occurrence (Hultmann et al., 2020; Meng et al., 2018). While the intergenerational transmission of individual forms of violence are generally well researched, qualitative studies with parents who were both exposed to IPV, including direct or indirect experiences such as witnessing, overhearing, or otherwise being aware of violence within the household, and experienced child maltreatment during their formative years are notably scarce. Hence, very little is known about whether and how co-occurrence itself contributes to intergenerational pathways. This is a notable gap given that co-occurrence is often described as a compounded risk factor (Hultmann et al., 2020) and it is known that such experiences often result in emotional dysregulation, insecure attachments, and learned behaviors that normalize violence (Oliveros & Coleman, 2021; Papalia & Widom, 2024; Zhang et al., 2023). Qualitative inquiry centers the voices and lived experiences of parents and would thus deepen our understanding of how parents interpret and assign meaning to their own childhood victimization of co-occurring IPV and child maltreatment. Parents’ subjective experiences and reflections are essential for informing the design, implementation, and evaluation of prevention and intervention strategies aimed at reducing violence within families.

This study set out to explore parents’ written narratives about their childhood victimization by both IPV and child maltreatment. The study employed qualitative methods and specifically, narrative analysis grounded in self-authored narratives, which allowed parents to articulate their own stories in their own words, to develop a nuanced exploration of victimized parents’ subjective experiences.

### The Intergenerational Cycle of Intimate Partner Violence and Child Maltreatment

In many families, children are exposed to multiple forms of harm simultaneously (Finkelhor et al., 2007). They may not only experience direct maltreatment, defined as acts of abuse or neglect that harm a child’s physical, emotional, or developmental well-being, but also witness violence between caregivers. Increasingly, IPV, which manifests as physical aggression, psychological abuse, and controlling behaviors, is recognized as a form of emotional maltreatment with psychological consequences as severe as those of direct abuse, even when the child is not the immediate target (Bogat et al., 2023).

Research indicates a significant overlap between children’s direct experiences of maltreatment and their exposure to IPV within the same household (Herbert et al., 2023; Mielityinen et al., 2024). Besides the fact that co-occurrence is common, its effects are broad, far-reaching, and often more severe than the outcomes of exposure to single forms of violence (Finkelhor et al., 2007; Hultmann et al., 2020). This cumulative exposure is associated with heightened risks of trauma symptoms (Cruz et al., 2022; Hultmann et al., 2020) as well as relational difficulties and parenting challenges (Lünneman et al., 2019; Taccini et al., 2025). It has also been found to intensify the risks of attachment disorganization, characterized by contradictory or confused behaviors in close relationships, often stemming from fear and lack of a consistent caregiver response (Cicchetti & Doyle, 2016), and increased vulnerability to future victimization and perpetration (Brown et al., 2021; Chen, 2024).

Research consistently shows that childhood exposure to violence increases the risk of both perpetrating and experiencing family violence later in life (Lünnemann et al., 2019; Madigan et al., 2019; van IJzendoorn et al., 2020). This process, known as the intergenerational transmission of violence (Haselschwerdt et al., 2017; Taccini et al., 2025), can occur through direct repetition of maltreatment (Madigan et al., 2019) or via an elevated likelihood of involvement in IPV as a victim or perpetrator (Shields et al., 2020) which constitutes intergenerational transmission if children are subsequently exposed to this violence. Meta-analytic evidence indicates that children of maltreated parents are nearly three times more likely to experience maltreatment themselves (Assink et al., 2018), and longitudinal studies link childhood maltreatment to increased IPV risk in adulthood (Curtis et al., 2023).

Several pathways may explain the intergenerational transmission of violence in families. The social learning perspective (Akers, 1998; Bandura, 1977) suggests that children exposed to violence in their families of origin may come to view these behaviors as acceptable ways to cultivate and maintain close relationships. Over time, these learned patterns can manifest in adulthood, either in violent parent–child relationships or in violent intimate partner relationships. Other explanatory frameworks emphasize the cumulative effect of trauma (Gélinas et al., 2025), suggesting that repeated adversities compound over time, disrupting emotional regulation and relational patterns that increase the risk of passing violent behaviors on to the next generation (Oliveros & Coleman, 2021).

Furthermore, child maltreatment and IPV have been consistently linked to insecure attachment (Baer & Matinez, 2006; Cicchetti & Doyle, 2016; Lo et al., 2019). Attachment theory offers a valuable framework for understanding how both childhood victimization and early relational trauma may be transmitted across generations. According to Bowlby (1969), attachment, which is shaped by the caregiver’s responsiveness to the child’s needs, forms the foundation for emotional regulation and a sense of safety for the child. Secure attachment figures help regulate emotional distress (Bowlby, 1969), whereas insecure attachment is linked to difficulties in emotion regulation. Individuals with attachment anxiety, marked by fear of rejection or abandonment, tend to be emotionally reactive, while those with attachment avoidance, characterized by discomfort with closeness and a tendency to suppress emotions, suppress their emotions and distance themselves from others (Conway et al., 2024; Domic-Siede et al., 2024).

Attachment disruptions often spill over into parenting, with insecure attachment linked to less sensitive and less consistent caregiving (Conway et al., 2024; Nichol et al., 2025). Disorganized attachment, characterized by contradictory or apprehensive behaviors rooted in unresolved trauma, is particularly associated with caregiving marked by fear and unpredictability (Baer & Martinez, 2006; Lo et al., 2019). Such patterns often interfere with parents’ reflective functioning, the capacity to understand behavior in terms of underlying mental states, which in turn undermines their ability to respond appropriately to children’s emotions (Fonagy & Target, 1997). When reflective functioning is compromised, the likelihood of dysfunctional parenting increases and, in some cases, may escalate to maltreatment.

Despite the vast body of evidence on the continuation of violence in families, there is also evidence that many parents with maltreatment histories do not maltreat their children (Greene et al., 2020; Haselschwerdt et al., 2017; Taccini et al., 2025); similarly, parents who have been exposed to IPV in their childhood homes do not always transfer these patterns into their partner relationships (Curtis et al., 2023). This evidence suggests that childhood victimization is only a single risk factor that may be efficacious alongside other risk factors. These other factors, such as parental stress and low paternal emotion recognition ability (Liel et al., 2022), may be equal in importance to or even more important than exposure to violence and can be independent predictors of a parental history of violence. Hence, the association between childhood victimization and later violence seems to be probabilistic rather than deterministic (Assink et al., 2018; Haselschwerdt et al., 2017), and protective factors implemented in a timely manner may moderate and prevent the consequences of childhood victimization (Meng et al., 2018; Taccini et al., 2025). Importantly, some individuals develop a secure attachment style achieved in adulthood despite insecure or adverse early experience (Filosa et al., 2024; Roisman et al., 2002).

Research on the intergenerational transmission of IPV and child maltreatment has largely used quantitative methods, thereby offering valuable insights into the likelihood of IPV and child maltreatment co-occurring within families and into how these individual forms of violence persist across generations (Madigan et al., 2019). These studies also identify key risk and protective factors that shape these trajectories (Liel et al., 2022; Meng et al., 2018). However, co-occurrence and its transmission are complex phenomena that are often overlooked in administrative datasets (Mielityinen et al., 2025) and difficult to capture through conventional quantitative approaches (Haselschwerd et al., 2017; Madigan et al., 2019). While qualitative studies exist (see e.g. Ciydem & Demirbilek, 2025; Haselschwerdt et al., 2017), they rarely explore how dual exposure is interpreted and transmitted across generations (Taccini et al., 2025) leaving it unclear whether and what role dual exposure plays in intergenerational transmission. Moreover, only few have centered parents’ own narrative voices although capturing subjective perspectives is essential for identifying perceived factors that contribute to perpetuation or disruption. Furthermore, the role of attachment, which is critical for emotion regulation and relational resilience for both children and adults, has received minimal attention, with existing research focusing mainly on parent–child attachment (e.g., Conway et al., 2024; Lo et al., 2019), overlooking its influence on intimate partnerships and co-parenting.

Narrative psychology offers a framework for addressing these gaps by examining how individuals make sense of their lives through internalized and evolving stories that link past experiences with present identities and imagined futures (McAdams, 2013). In McAdams’s theory of narrative identity, these stories serve as meaning-making structures through which people interpret adversity, coherence, and change over time (McAdams & McClean., 2013). This perspective highlights how individuals position themselves in relation to earlier experiences such as childhood maltreatment and exposure to IPV and how they derive explanations for continuity, rupture, or transformation across generations. As such, narrative psychology provides a conceptual lens for capturing subjective interpretations that quantitative approaches often overlook.

This study fills gaps in the current literature by examining how parents who have experienced both child maltreatment and exposure to IPV in childhood narrate intergenerational transmission. To address this aim, the study asks: How do parents with childhood experiences of both maltreatment and exposure to IPV narrate and make meaning of intergenerational continuity or change in violence? The study examines the interpretive and meaning-making processes through which individuals position themselves in relation to their pasts, particularly the meanings they attribute to continuity and change across generations. By analyzing self-authored written narratives, the study accesses forms of reflexivity and self-presentation that may not emerge in dialogic settings. This approach offers a nuanced understanding of both the vulnerabilities that perpetuate violence and the strengths that foster resilience and cycle-breaking behaviors, thereby providing new perspectives to encourage prevention and intervention.

## Data and Methods

The data examined in this study are a set of written narratives from Finnish parents with histories of both physical maltreatment and physical IPV between their parents in childhood. An open call was issued inviting parents to share their experiences of physical violence in their childhood and current families. Although the primary focus was experiences of physical violence in both childhood and adulthood, the participants were not discouraged from describing experiences of psychological violence. This approach acknowledges that psychological violence is often inherent in situations of physical violence (Hart et al., 1996) and that inquiries about physical violence frequently elicit narratives that also reveal psychological harm. The invitation included a set of background questions designed to gather details about the parents’ relationships with the family members who experienced violence. These background questions were optional, allowing the participants to focus on their narratives since in narrative research the focus is on meaning‑making, not sample uniformity (Riessman, 2008).

Narrative prompts were crafted to encourage the caregivers to share their stories in detail. Because the study sought to explore both the continuation and the cessation of violence across generations, two types of narrative prompts were necessary: one directed at participants who experienced ongoing patterns of violence in their current family life, and another designed for those who perceived themselves as having broken one or both cycles of violence. These prompts were as follows: “How do you perceive the connection between the experiences of violence in your childhood family and the physical violence occurring in your current family?”, “What factors do you think are associated with the continuation of violence from your childhood family to your current family?”, “If you have managed to break one or both cycles of violence, what do you think is behind your ability to avoid continuing the violence yourself?” and “How do you perceive that your experiences of violence in your childhood family are related to the absence of violence in your current family?”.

The data were collected between October 2024 and March 2025 using two secure platforms: Penna, which is part of the Finnish Social Service Data Archive, and LimeSurvey, a free and open-source online survey application. The link to these platforms was shared on social media (Twitter, Facebook, and Instagram) through both professional and personal networks. Caregivers were also recruited via national newspapers and noticeboards. All parents (biological or non-biological) older than 18 years old were invited to write. This resulted in a self-selected sample of 15 parents and their narratives which varied in length. The total length of the text was 20 pages (Times New Roman, 12‑point font).

Eight of the 15 parents reported their ages, which varied between 25 and 64 years. Eight of the parents were female, and the rest did not report their gender. Nine of the parents reported living in a nuclear family (spouses and their mutual children), one in a single parenthood family (one caregiver and children), and two in blended families (two spouses, not all children are mutual), while one parent reported their current family status being something other than these previously mentioned. This left two parents who did not specify their family situations. Information on nationality was not requested, but all the narratives were written in Finnish.

The study was approved by the local ethics committee (approval number 30/2024) and adhered to the ethical guidelines of the Finnish National Board on Research Integrity. The researchers introduced the study and stipulated the right to withdraw at any point in the research advertisement, reiterating this right in writing before the parents submitted their narratives. Consequently, informed consent was obtained from all participants, and they were assured that their responses would be used solely for research purposes. Penna and LimeSurvey are secure platforms that ensure the confidentiality of participants.

The data were stored in the first author’s personal network file behind two-phase authentication. All identifying information (names, addresses, etc.) was removed before storage and analysis. Since the narratives were anonymous, the researchers were not obliged or able to report the cases of child maltreatment to authorities. Finally, several support channels were provided at the end of the invitation for narratives, which parents could contact if they experienced any emotions or issues they wished to discuss further after writing.

The research team engaged in active reflexivity throughout the study, recognizing that the interpretation of narratives is shaped by researchers’ positionalities and analytic roles (Soedirgo & Glas, 2020). The first author was primarily responsible for data analysis, as well as for developing the methodological approach and reporting the findings. This central role in constructing the composite narratives required continuous critical reflection on how personal assumptions, theoretical orientations, and prior knowledge of intergenerational violence might influence analytic decisions and interpretations. Particular attention was paid to preserving participants’ voices when constructing composite narratives, balancing interpretive abstraction with fidelity to the original accounts.

Reflexivity was practiced iteratively during the analytic process through memo writing and ongoing self-reflection, particularly in relation to how patterns of continuity, disruption, and responsibility were identified and interpreted. To support analytic rigor, emerging interpretations and narrative constructions were regularly discussed with co-authors, who contributed alternative perspectives, critical questions, and conceptual clarifications. This collaborative process helped to challenge taken-for-granted assumptions and ensured that the analysis remained grounded in the data rather than in a single interpretive lens.

### Data Analysis

This study draws on concepts from the narrative work of Riessman (2008), Wertz et al. (2011), Willis (2019), and Johnston (2024). Building on Riessman’s interpretive stance, the analysis focuses on how storytellers make sense of their pasts rather than merely reproducing events, attending to the form, function, and meaning of their narratives. As researchers, we carry an ethical responsibility to re‑represent participants’ voices with care while conveying the richness and complexity of their perspectives. In line with narrative psychology, which conceptualizes personal stories as evolving meaning‑making structures through which individuals interpret their pasts and position themselves across time (McAdams, 2013; McAdams & McLean, 2013), this approach emphasizes how narrative constructions articulate identity, agency, and perceived continuity or change. The use of composite narratives (Johnston, 2024; Wertz et al., 2011; Willis, 2019) further supports this interpretive focus by integrating multiple accounts into shared storylines while preserving narrative flow and complexity. Together, these narrative approaches support an analysis attentive to how parents construct meaning, position themselves across time, and narrate perceived continuity or change in the intergenerational transmission of violence.

**Fig. 1:**
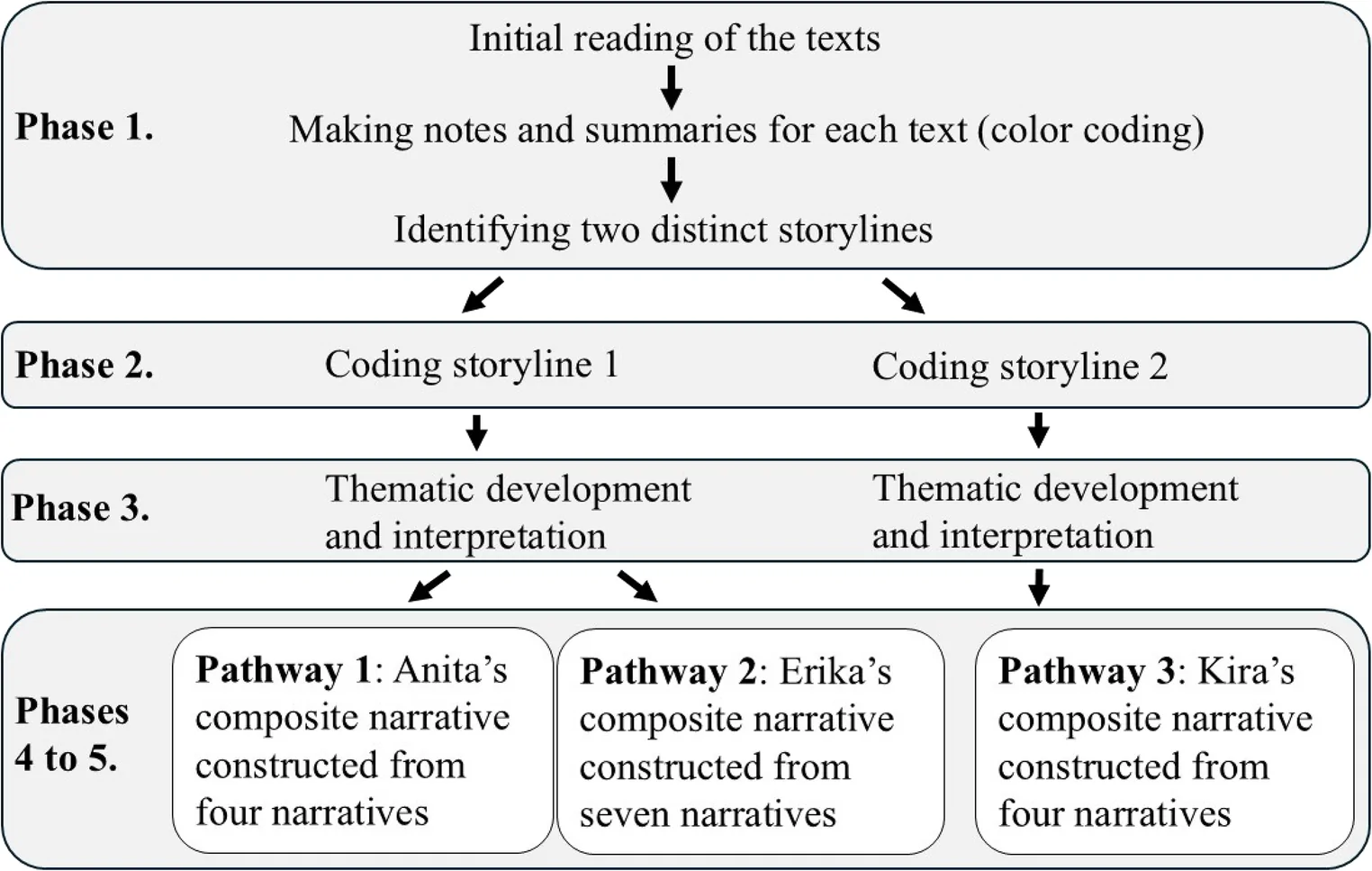
Analysis process

The analysis followed several key steps illustrated in Fig. 1.

#### **Phase 1. Initial Reading and Familiarization**

First, the narratives were transferred to Atlas.ti version 24. We assigned the narratives numbers and different colors to keep track of them during the analysis process. The first step included reading the narratives multiple times to become familiar with the content and gain an in-depth understanding of how the parents narrated their experiences. Through this first phase, two distinct storylines were identified: (1) narratives in which the intergenerational cycle of violence had been broken, and no violence (either IPV or child maltreatment) had ever occurred in the parent’s current family (11 narratives), and (2) narratives in which the intergenerational cycle of child maltreatment had continued, and the parent had maltreated or was still maltreating their children (4 narratives). As stated before, none of the parents wrote about experiencing or perpetrating IPV in their current families. From this stage, the narratives were divided and analyzed according to these distinct storylines.

#### **Phase 2. Open Coding**

The next step involved open coding with both descriptive and structural codes, applied separately to each storyline but using the same procedure. The primary analytic focus was the content of the narratives—what parents wrote about intergenerational transmission and the factors they perceived as contributing to its continuation or disruption. Because the narratives centered on early experiences and present‑day parenting, temporality naturally became central, drawing attention not only to what was recounted but to how parents sequenced events, positioned themselves in relation to their past, and constructed meaning across time. In line with the narrative‑psychological perspective, the analysis therefore attended to both content and form: what happened, and how the story was authored to make sense of that experience. The guiding questions included: How do parents position themselves in relation to their past experiences? What meanings do they assign to those experiences in the context of intergenerational transmission? How do they describe continuity or change across generations? And what individual, relational, or societal factors contribute to either continuation or disruption? Meaningful segments of text were assigned inductive codes to ensure that the parents’ voices guided the analytic process.

#### Phase 3. Searching Themes

After this initial coding, we added all codes to a network to visualize their distribution. Since two distinct storylines had already been detected, the codes from stories in which the cycle had been broken were added to one network, and codes from stories in which maltreatment had continued were added to the other. This step shifted the analysis from coding to thematic development and interpretation. The idea was to look for shared experiences and patterns that resonated across the narratives inside the storylines and to examine whether the individual stories became collective ones.

#### Phase 4. Identifying Narrative Pathways

The thematic analysis revealed several recurring themes across the narratives, showing how particular structures and patterns were expressed in different accounts. Within the eleven narratives describing the breaking of the intergenerational cycle of violence, two distinct narrative pathways emerged. Five narratives depicted early, conscious moral agency and a relatively effortless rejection of violence, while six portrayed the sustained effort required to parent against one’s past, accompanied by emotional, cognitive, and relational transformations. Narratives describing the continuation of maltreatment formed a single pathway. Altogether, these analyses resulted in three narrative pathways.

#### Phase 5. Constructing Composite Narratives

After establishing the three pathways, the analysis returned to the original texts and the codes associated with each pathway to deepen interpretation and build a composite narrative for each group. These composites synthesize key elements shared across multiple accounts and present fictionalized protagonists grounded in the data. They do not represent any single participant but instead capture the core themes, narrative tone, and identity shifts characteristic of each pathway. Because Phase 4 relied on open coding and thematic comparison, the composites were directly linked to the patterns identified in the earlier phase, ensuring a transparent connection between the source narratives and the final constructions. Although the narratives varied in length, the composite narratives were grounded in their substantive content rather than their formal uniformity. Care was taken to ensure that no longer narrative disproportionately shaped the account simply by virtue of containing more material. Each original narrative contributed thematically, and the core meanings within shorter accounts were equally incorporated into the analysis. All quotations are drawn verbatim from parents’ narratives, with the only transformation involving the integration of material from multiple accounts into a single storyline. Quotations were translated into English using the European Commission’s eTranslation program. For narrative clarity, the pronoun “she” is used in the third‑person composites; this is a narrative convention and does not reflect participants’ actual genders. Each composite begins with a brief description of the individual narratives on which it is based, and the themes characteristic of each pathway are presented in Supplementary Tables 1, 2 and 3.

## Results

None of the parents wrote about physical IPV in their current families. Eleven narratives described journeys through which parents had managed to break the cycles of both maltreatment and IPV. Four of the narratives described journeys in which parents had directed or were still directing physical maltreatment toward their children.

Three composite narratives were created from the data. They illustrate the different types of narratives that parents who experience both child maltreatment and IPV in their childhood construct to articulate the intergenerational transmission of violence. We refer to these composite narratives as pathways not only in accordance with methodological convention but also because of the data itself, which indicated that the parents’ narratives unfolded as distinct journeys, each representing a different trajectory. The term “pathway” is used to emphasize their processual nature and illustrate the mutual differences between them.

### Pathway 1: Anita’s Early, Conscious Decision To Parent Differently – “I Decided To Be Different”

Anita’s pathway is a composite construction that synthesizes four individuals’ narratives. In each narrative, the parents had successfully broken both cycles, and there was no maltreatment or IPV in their current families. These parents had experienced physical punishments in their own families, such as pinching, mouth washing, and hitting with straps. Neglect surfaced in moments when their caregivers left them alone or emotionally retracted from them. Parental conflicts included screaming, insulting, and occasional physical altercations.

For Anita, the rejection of violence was a clear, early, and conscious decision rather than a gradual journey. For her, this stance was final and self-contained. Therefore, her identity was firmly constructed early in life, before becoming a parent, and clearly in contrast to her own victimization. She wanted to act in direct contrast to how she was raised: “I decided already when young, a teenager, that I don’t want the kind of life that my childhood and adolescence had been. . It was clear that I would do things quite differently than my parents did. It would be strange to be violent.”.

Anita felt that her commitment to non-violence was an intrinsic part of her identity, an innate orientation rather than a learned or hard-won transformation. She narrated how she had always felt that she was nonviolent and always known that violence was wrong. Anita linked this awareness to early experiences of emotional regulation, sensitivity to pain, and a temperament that resisted rage. She had transferred this early learned emotional regulation to her own parenting, which was characterized by nonviolent commitment: “Maybe it’s also about having the ability to pause between emotion and action, so that the impulse doesn’t immediately turn into behavior. . I’ve had to tell him [my child] many times not to hit, explaining that it hurts. And I’ve often had to help him regulate strong emotions to prevent them from erupting physically toward others.”.

Anita narrated her childhood with emotional restraint. Her account of victimization reads more like a factual inventory than an emotionally charged recollection: “As a child, I experienced hitting with straps, washing my mouth with soap, hair pulling.” Similarly, when describing neglect and exposure to IPV, she maintained a descriptive tone. Thus, rather than expressing trauma, Anita articulated a clear awareness that her upbringing was emotionally and ethically deficient. Nevertheless, she continued to maintain a close relationship with her parents: “I would have liked her to apologize for what she did. It hasn’t caused trauma. I’ve always had a good relationship with my parents.”

Anita made a conscious decision to become a parent later in life. This was a reflective choice for her, one that encouraged emotional maturity and alignment with nonviolent values: “I read extensively on parenting-related literature and sought both peer support and professional assistance from maternity and child health clinics as well as family counseling services.”

Anita presented herself as an active agent who was in control of her own life. Although she received early relational support from siblings and grandparents while constructing her nonviolent identity during her formative years, this was to buffer her resilience, and she did not rely on these significant others. Along the same lines, female teachers offered a new model of womanhood for her: “I had several protective factors in my childhood: wonderfully loving grandparents and an older half-sister. In addition, I have encountered wonderful female teachers who modeled a different kind of womanhood. I got compliments from them, and my self-confidence was strengthened.”.

When it was time to start her own family, Anita deliberately chose safe relationships in terms of both her spouse and her spouse’s family. Anita built partnerships on gentle, respectful dynamics, contrasting them with her own upbringing: “My spouse comes from a completely different, normal, and safe family, which has helped to build a safer life for our family as well. My mother-in-law acted as an example, and I was able to see a gentler way to act.”.

Anita was acutely aware of the intergenerational nature of violence. Rather than narrating violence as isolated or purely personal, Anita contextualized it as part of a continuum of inherited harm and cultural continuity, a tradition shaped by rural disciplinary norms and post-war societal conditions. She wrote, “Both of my parents had experienced severe disciplinary violence as children in the countryside after the war.” However, instead of solely blaming these historical and cultural norms, she wrote that the violence she experienced as a child was attributable to her parents failing to raise her properly: “Already as a child, I thought that it [violence] is parental incompetence and repetition of my mother’s own history.” Unlike her parents, who simply transferred violence as a learned means of bringing up children, Anita took control and broke the cycle of maltreatment. She expressed being proud of her own parenting and observing her own child becoming a sensitive parent, and seeing this reflected in the upbringing of her grandchildren, reinforced Anita’s sense of legacy and success, especially in contrast to the violence and emotional neglect she experienced in her childhood: “Now it’s time to follow my first grandchild and my child’s sensitive parenting toward my grandchild. He has also reflected on the parenting he received and practices his own style of tender, gentle, yet firm upbringing.”.

### Pathway 2: Erika’s Ongoing Struggle Under the Weight of the Past – “I’m Doing Conscious and Constant Work to Parent Differently”

Erika’s pathway is a composite construction assembled from seven individual narratives. In all of them, the parents had managed to break both cycles, and neither maltreatment nor IPV was present in their current families. These parents experienced severe acts of physical violence, such as beating, strangling, and hitting with straps. These acts of psychological violence and exposure to both emotional and physical IPV played a significant role in creating a pervasive atmosphere of fear, instability, and emotional distress.

For Erika, breaking the cycle was an ongoing process which took constant effort to parent against the past. She highlighted the emotional and cognitive labor involved in unlearning harmful patterns and rejecting the parenting models she observed in her childhood: **“**Breaking the cycle has demanded conscious effort, thinking, and learning. I’m not saying that the decision to restrain from physical discipline has always been easy. I have decided to be a better parent than I had.”. Moreover, this meant that she had to actively reflect on her past experiences, consciously recognize risks, and choose self-control. Erika narrated how she “had the temptation to grab [her] raging toddler by the hair” and how she learned to retreat in these situations: **“**When I receive anger from my children, I notice that it provokes anger in me as well. In these situations, I retreat. The most important thing has been to recognize those risky situations, stop myself, and find another way to act. I would emphasize that it would have been much easier to follow the parents’ example, as it comes instinctively—like a reflex, without thinking.”.

The emotional atmosphere of Erika’s childhood home, characterized by hypervigilance and emotional insecurity, shaped her internal emotional world as a child. She refers to “being like a coiled spring” and explains how she could never be sure why her mother or father was angry at her or what she had done wrong this time: “If there was no cause for “discipline,” then it was invented by [my] father. Sometimes, we could already hear the volume of sound rising from our own rooms. At that point, we started to quietly clean the room so that there would be as little shouting and stuff to throw at us as possible. But it didn’t help.”. Childhood insecurity was further reinforced by coerced emotional suppression, and she recounted being forced to hide her feelings and emotional reactions: “My father couldn’t stand the useless crying. He said he would give us the right reason to cry if he thought we were crying for nothing. I was punished for showing negative emotions. I learned not to react until I was in a safe situation.”. In addition to the direct emotional maltreatment in her childhood home, Erika remembered the emotional and physical IPV that further intensified the atmosphere of fear and anxiety: “Something broke inside me when I saw my mother hit my father on the head with a beer bottle, and it broke upon hitting. I have never been that afraid. It was like the bottom of my world had fallen out.”. These situations were sometimes contributed by alcohol and financial hardship. Erika also recognized that violence was often hidden from others: “My father told me to go to another room and slapped a smile onto my face. We had to go back to the living room as if nothing had happened.”. She was sometimes intimidated into silence by child welfare.

Erika narrated how emotional coldness and distance persisted into adulthood, raising concerns about her capacity to provide physical and emotional affection to her own children: “Sometimes I feel like a cold mother. I’m afraid my children need more physical affection than I can give them.”. Erika went to therapy to deal with these concerns. She also sought help from the maternity and child health clinic as she was afraid that she would treat her children with anger and roughness since this was the way her parents had reacted: “Even though the child’s crying is distressing, I try to understand and take him in my arms. I’ve never experienced it from my parents, so I’ve had to learn all the skills myself.” She intentionally used practical actions and parenting techniques as both protective strategies and reparative strategies, especially when her firstborn was a toddler with a fierce temperament: “I am not sure whose nerves this [holding an angry child] affected more: mine or my child’s. In part, those moments probably healed me, too.”. Additionally, Erika intentionally selected an emotionally safe partner who shared same values and parenting practices which were based on reflection, emotional responsiveness, respect for the child’s autonomy, boundaries without intimidation, and emotional labor.

In adulthood Erika navigated between bitterness and family bonds, as she had a close relationship with her parents, although she had not reached full closure: “I have almost let go of the bitterness from my childhood experiences and my mother’s (and father’s) actions, but I have not forgiven them. I believe I have the right to be angry. An apology from my parents would help, it might allow me to feel truly seen.”. Erika had a nuanced understanding of how disciplinary violence was embedded in a broader sociocultural heritage and she referenced the Finnish proverb “He who spares the rod hates his child“ to contextualize the disciplinary practices of her parents’ generation. Erika also noted how violence extended into sibling relationships in her childhood family: “I myself became violent toward my siblings as a sensitive child because of my childhood experiences of violence, since we were left to solve our quarrels using fists.”

Erika was extremely proud of her ability to interrupt the cycle of violence and the harmful parenting practices despite the struggle it required. She admired her own achievements and ability to offer her children the childhood she would have wanted and deserved: “For example, when I listen to my children’s giggles and screams, watch them express their disappointment about something, or clean up their tracks in the living room or kitchen, I think, I did it. I have succeeded.”.

### Pathway 3: Kira’s Progress Toward Breaking the Cycle After Perpetuating It – “I Have Done What My Parent Did”

Kira’s pathway is a composite account constructed from four narratives. These parents had used or were using physical maltreatment on their children, hence continuing maltreatment between generations. They had all broken the transmission of IPV. These parents experienced both disciplinary violence, such as spanking and hair pulling, and severe maltreatment, such as beating with leather belts and hitting. Emotional violence and insecurity further destabilized their home environments. IPV was mentioned only briefly.

Kira balanced her childhood trauma with the violence that she transferred to her current family. This way, for Kira, her childhood experiences were not just background incidents; they were foundational to her entire pathway into adulthood and imposed a heavy emotional burden: ”I recognize that this same shadow and darkness have always been inside me, as a child, a big and dominant burden that shadowed all my life. Fading now that I’m an adult and working on it. But ready to act if necessary. Mental wounds never heal, and I’m still carrying the burden because of what my parents did.”.

Kira’s childhood was characterized by emotional suppression. She explains how she had to hide her emotions to avoid violence as well as the hurt the violence caused. Now, as an adult, she was facing her childhood emotions: “As a child, I wasn’t allowed to express my own feelings. All of it had to be pushed deep inside. Hidden. Now, I can begin to uncover it, face it on my own, and gently care for my inner child.”. Furthermore, Kira experienced neglect and insecurity in her childhood: “I was abandoned home alone, and I was left in the understanding that my mother wasn’t coming back at all.” She later adds, “I often had to go to the tree at my grandmother’s house and pick a birch for my mother. The whip was always by the kitchen doorway; it was used to threaten.” Threatening was a common way to control children in her childhood family.

Kira struggled with control and emotion regulation in her adulthood. She was constantly trying to learn nonviolent coping strategies and anger management in situations in which she felt her anger rising: “I try to target this darkness, rage, and anger so that it does not target my children. I tried to restrain the feeling enough to place the child on the floor or on the couch, and then I would throw objects instead. I defuse my anger by beating my hands on the floor and screaming.” She questioned these alternative methods herself: “Do my children find it [beating the floor] frightening at times? Probably. Does it traumatize them? Possibly, to some extent.” Yet, she tried to verbalize her emotions and actions to her children so they would understand that her rage was not their fault.

Despite the effort to restrain her own violence, Kira sometimes resorted to the same harmful patterns her parents used. She slapped her children, pulled their hair, and pushed them. Kira clearly saw the association between her actions and the violence she experienced in her childhood: “I often recognize the situation where my parents would have done something. Once, I ended up doing the same thing my father did.” Kira also recognized how her emotional response to her child’s cry was affected by her childhood experiences: “I had this feeling inside me that ‘you can’t cry either, because I wasn’t allowed either.’”.

The physical maltreatment usually occurred when she felt the pressure had become overwhelming: “… eventually some situation with a defiant child has escalated into violence.” In addition to physical violence, Kira had yelled and threatened to abandon her children. When one of her children was a baby, Kira remembered having uncontrollable outbursts of rage.

Kira’s reaction to these violent incidents was usually remorse and fear: “Then I cried and apologized to the child. I deeply regret it. It always scared me.”. However, Kira also minimized some of her actions by saying that she did not use violence constantly but only occasionally. She also emphasized that she had never done anything that would have left a mark or hurt the children very much.

Kira recognized some turning points along her pathway to breaking the cycle of maltreatment. One of these turning points was when Kira’s mother-in-law questioned her way of shouting at her children: “Another adult said and told me it wasn’t normal, and it was wrong. I asked for help and broke the cycle of violence.”. She also recognized how her children’s resistance to obeying forced her to learn new approaches, and she emphasized the ongoing journey of healing: “I have no other choice but to continue the work I’ve started. The road is long and rocky, and I’m still taking care of myself.”

Although Kira’s pathway was still ongoing, she felt optimistic about her and her children’s futures. She also expressed gratitude for the opportunity to learn nonviolent parenting practices, framing it as both a personal achievement and a relational turning point: “Nowadays, I am happy and grateful that I have had to learn nonviolent behavior. I have a healthier relationship with my children than my parents have with me. I hope that I can lighten this burden so that my own children will have a slightly better chance of breaking the cycle of trauma.”:

## Discussion

This study set out to explore how parents with childhood experiences of both maltreatment and exposure to IPV narrate and make meaning of the intergenerational transmission of violence. Grounded in narrative psychology, the analysis demonstrates how parents’ meaning‑making, temporal positioning, and identity construction illuminate both the vulnerabilities that sustain intergenerational patterns of violence and the strengths that facilitate resilience and cycle‑breaking (cf. McAdams, 2013; McAdams & McLean, 2013). Further aligning with narrative psychology, the narratives show that intergenerational processes unfold not only through behavior but also through the stories parents tell about themselves and how they interpret the past, position the present self, and imagine possible futures (Riessman, 2008).

The 15 parents who participated in this study described intergenerational violence in three distinct narrative pathways. Parents on the first and second pathways managed to break free of both cycles: IPV and child maltreatment. Parents on the third pathway continued the cycle of maltreatment but broke the cycle of IPV. This division supports previous evidence that not all who experience violence in their formative years perpetuate it in adulthood (Curtis et al., 2023; Greene et al., 2020). However, as child maltreatment was transferred in four narratives, the findings also underscore that along with other known risk factors (Liel et al., 2022) childhood victimization remains a predictor of the persistence of child maltreatment across generations (Madigan et al., 2019; van IJzendoorn et al., 2020). Prevention and intervention efforts should therefore continue to emphasize this risk while also attending to how parents interpret and integrate their early experiences into current parental identities.

The findings challenge simplified models of intergenerational transmission by showing that discontinuing IPV does not automatically end child maltreatment. While previous research has emphasized that experiencing childhood maltreatment and exposure to IPV increases the likelihood of IPV in adulthood (Assink et al., 2018; Curtis et al., 2023; Shields et al., 2020), the narratives here suggest more nuanced pathways in which corrective relational experiences, such as intentional partner choices, can reduce the risks of both IPV and child maltreatment. These choices may reorganize attachment and foster earned security (Bowlby, 1969; Iyengar et al., 2019; Roisman et al., 2002), interrupting transmission. At the same time, it is important to interpret the emphasis on partner choice with caution. IPV is not always predictable, and relationships that are initially non-violent may become violent over time. Framing the absence of IPV primarily in terms of individual partner selection risks implying responsibility for avoiding violence. Rather, these narratives may reflect a combination of intentional decision-making, relational dynamics, and contingent factors beyond individuals’ control. Although IPV and child maltreatment frequently co-occur (Herbert et al., 2023; Mielityinen et al., 2024), the complexity highlighted in these narratives also underscores that they involve different dynamics, requiring tailored interventions alongside a holistic perspective. Because most previous studies have examined IPV and child maltreatment separately, the distinct pathways revealed here highlight the analytical importance of studying dual exposure as a unique intergenerational context.

Drawing on self-authored narratives, a method rarely employed in research on intergenerational transmission, this study foregrounds parents’ own interpretive frameworks, extracting insights into how they make sense of violence and its discontinuation rather than imposing externally defined categories (Johnston, 2024; Riessman, 2008; Wertz et al., 2011; Willis, 2019). Anita’s pathway illustrates an early authorial stance: a deliberate identity commitment to reject violence and parent differently, forged before parenthood and narrated with relative ease and coherence. Erika’s pathway illustrates ongoing re-authoring as childhood pain often resurfaces during challenging parenting moments, requiring intentional self-regulation and reflection to resist internalized patterns (Gélinas et al., 2025; Greene et al., 2020; Taccini et al., 2025). The ability to pause between emotional provocation and response reflects mentalization and earned secure attachment (Fonagy & Target, 1997; Roisman et al., 2002), capacities that are remarkable given her early environment marked by fear and emotional suppression.

For Erika, caregiving became both protective and reparative, supported by strategies such as therapy, deliberate partner selection, and comfort-oriented parenting practices. These corrective relational experiences facilitate reorganizing attachments, shifting from insecurity to security, and interrupting the transmission of trauma (Filosa et al., 2024; Nichol et al., 2025). Attachment-related vulnerabilities further compound these challenges. Insecure attachment styles are associated with less effective regulation under stress (Domic-Siede et al., 2024), and meta-analytic evidence confirms that insecure adult attachment significantly increases the risk of child maltreatment (Lo et al., 2019). Research consistently shows that maltreatment-related attachment insecurity mediates the link between early trauma and later violent behavior (Cicchetti & Doyle, 2016; Meinck et al., 2025; Papalia & Widom, 2024). These patterns not only hinder emotional attunement but also perpetuate relational dynamics characterized by avoidance or disorganization (van IJzendoorn et al., 1999), reinforcing the intergenerational transmission of violence.

Kira’s pathway illustrates how maltreatment can persist across generations, instantiating mechanisms described by social learning theory (Akers, 1998; Bandura, 1977) and documented in longitudinal research (Shields et al., 2020) as well as in recent realist review (Meinck et al., 2025). Harmful behaviors previously endured are repeated when efforts to manage emotional triggers and regulate behavior falter, often under stress, underscoring why deficits in social–emotional competencies remain among the most significant risk factors for intergenerational maltreatment (Corso et al., 2025). Through therapy and interventions Kira acquired emotion regulation skills that helped her translate her parenting intentions into consistent practices.

Together, these pathways demonstrate that there is no universal model for preventing the intergenerational transmission of violence. This variability in trajectories aligns with recent evidence emphasizing that intergenerational transmission of violence operates through context-dependent mechanisms and relational processes, leading to both continuity and disruption across generations (Meinck et al., 2025; Woollett et al., 2025). Instead, prevention and intervention efforts must be tailored and Erika’s experiences underscore how preventive efforts must address the whole family, strengthening attachment, teaching parenting skills, and cultivating coping mechanisms to sustain nonviolent parenting (Backhaus et al., 2023; Bernard et al., 2012). Parents like Kira constantly struggle to disentangle themselves from past trauma, indicating they require sustained support across multiple stages of parenthood. This highlights the importance of preventive measures, such as therapy and guidance from healthcare professionals, particularly in child health clinics, as well as timely interventions (Dierkhising et al., 2019; Meng et al., 2018).

Beyond behavioral strategies, this study introduces a narrative-psychological dimension. Kira exemplifies how parents who perpetuate maltreatment referred to their childhood exposure to IPV only briefly, suggesting that they may not have assigned significant narrative meaning to it, while those who broke the cycle recalled it as emotionally significant. This suggests that co-occurring forms of violence can be behaviorally linked but psychologically compartmentalized. Where exposure is acknowledged and narratively integrated into a coherent life story, reflective functioning i.e. the capacity to understand one’s own and the child’s behavior in terms of underlying mental states (Fonagy & Target, 1997) and earned secure attachment (Filosa et al., 2024; Roisman et al., 2002) appear more likely to develop, thereby reducing risk. Thus, the critical factor may not be mere exposure but the extent to which relational traumas are recognized, emotionally processed, and integrated into a coherent attachment narrative (see also Woollett et al., 2025), a process consistent with evidence that attachment insecurity mediates links between early maltreatment and later relational violence (Cicchetti & Doyle, 2016; Papalia & Widom, 2023). These difficulties often stem from developmental histories marked by neglect, emotional suppression, and threatening behavior, which undermine secure attachment and effective regulation (Baer & Martinez, 2017; Gélinas et al., 2025; Lo et al., 2019; Woollett et al., 2025).

At the same time, parents often situate their experiences within a broader sociohistorical context, recognizing that their own parents were also victims of maltreatment, an experience normalized and socially accepted during their childhood. These reflections show how personal narratives are embedded within broader sociohistorical storylines, highlighting the cultural dimension of narrative identity (McAdams, 2013). Together, these findings underscore that intergenerational trauma manifests through complex behavioral and psychological pathways (Greene et al., 2020), requiring preventive actions and interventions that both support safe partner selection and enable retrospection and reflection on childhood victimization which might help parents to symbolize, organize, and re‑author traumatic experiences.

### Limitations and Further Directions

Some limitations of this research should be identified. First, although the narratives were rich in content, contextual details were obtained through optional questions and the available information varied across participants. This inconsistency limits the comparability of narratives and the descriptiveness of the sample, although such variation is common and acceptable in narrative research where meaning‑making, rather than demographic completeness, is central. Moreover, since the eight parents who reported their gender were female, further research should seek to deepen our understanding of fathers’ perspectives, especially as the parents in this study emphasized the spouses’ roles in creating safe environments for themselves and their children. Second, the study’s focus on physical violence may narrow the range of intergenerational patterns identified, as it does not fully capture potential continuities in non‑physical forms of violence, such as emotional or psychological abuse. In addition, none of the participants reported involvement in IPV in their adult relationships, which limits the extent to which the findings can speak to pathways of ongoing intergenerational transmission of IPV, instead highlighting processes of risk negotiation and potential interruption.

Third, traumatic or emotionally charged events may be recalled inaccurately, particularly when shaped by unresolved trauma or defensive processing. This underscores the importance of incorporating children’s perspectives, as they are actively living within these family dynamics and may offer more immediate and contextually grounded insights into experiences of intergenerational violence. Furthermore, narratives reflect what participants choose to share, and they may omit or distort experiences. The accounts in this study also largely reflected orientations toward breaking or interrupting cycles of violence, suggesting possible self-selection bias and limiting insight into trajectories where violence is normalized or unchallenged.

Finally, parents may present themselves as competent or morally responsible, downplaying harmful behaviors. However, this is more likely to happen in interview settings, where some individuals may struggle to share their experiences face to face due to shame, cultural norms, or being emotionally overwhelmed. A key strength of this study was the narrative approach, which allowed the parents to reflect more freely and deeply on their stories, often revealing contradictions, emotional complexity, and key turning points. This also enabled the parents to narrate experiences of emotional maltreatment, which are usually key elements in physical violence; however, in this context, they were recounted by the parents’ own will. This underlines the need to broaden our understanding of intergenerational transmission to include the emotional dimensions of trauma, which are often less visible but deeply impactful.

## Conclusion

This study reinforces prior evidence that the intergenerational transmission of violence is probabilistic rather than deterministic, while simultaneously highlighting that childhood victimization remains a significant risk factor for subsequent perpetration of child maltreatment. Additionally, the findings indicate that intergenerational transmission is shaped not only by exposure to co-occurring forms of violence but even more by how these relational traumas are understood and integrated into a coherent attachment narrative. When both maltreatment and IPV are acknowledged and meaningfully incorporated into this narrative, individuals can work toward earned secure attachment, both as parents and partners. This process enables the repair of parenting practices, and fosters a safer, more nurturing environment for the entire family. For some, insecure attachment patterns formed early in life make daily efforts to balance stress and regulate emotions overwhelming, leading them to resort to violence and repeat the harmful behaviors they once endured. Hence, the study’s implications for practice point to a dual approach. On the one hand, prevention and intervention efforts targeting behavioral change are crucial. These include spousal and professional support as well as strengthening parenting and emotion‑regulation skills. On the other hand, internal motivation and parents’ capacity and opportunity to re‑author their childhood experiences can help interrupt, and ultimately end, the intergenerational transmission of violence.

## Supplementary Information

Below is the link to the electronic supplementary material.

Supplementary Material 1

## Data Availability

Metadata are available upon reasonable request from the corresponding author.

## Declarations

### AI Statement

Microsoft Copilot was used to assist with language editing of the manuscript, including improvements to grammar, clarity, and readability. All scientific content, interpretations, analyses, and conclusions were developed and verified by the authors.
