# Supplementary material for: Narratives about Breaking the Cycle of Intergenerational Violence in Families: Different Routes, Same Goal
Source: J Child Adolesc Trauma. 2026 Aug 11;19(4):2445–58. doi: 10.1007/s40653-026-00961-9 (PMC13737356; doi:10.1007/s40653-026-00961-9)
Supplement: Supplementary file 1 [file 40653_2026_961_MOESM1_ESM.docx]

**Supplementary table 1.** Main themes and subthemes behind pathway 1

| **Pathway 1** | **Main themes** | **Subthemes** |
| --- | --- | --- |
|  | Conscious Moral Agency and early Nonviolent Commitment | Early conscious decision |
|  |  | Innate orientation toward nonviolence |
|  |  | Emotional regulation |
|  |  | Deliberate rejection of violence |
|  | Early Relational and Institutional Resources for Resilience | Positive influence of other females |
|  |  | Choosing safe relationships |
|  |  | Studying social sciences |
|  |  | Early relational support |
|  |  | Seeking and applying support |
|  | Sociohistorical Context of Violence | Pride and accomplishment in own parenting |
|  |  | Inherited harm and cultural continuity |
|  | Ambivalence in Parental Relationships | Seeking accountability and emotional boundaries |
|  |  | Minimization of harm and emotional closeness |

**Supplementary table 2.** Main themes and subthemes behind pathway 2

| **Pathway 2** | **Main themes** | **Subthemes** |
| --- | --- | --- |
|  | Constant Effort to Parent Against the Past | Conscious work and effort to parent differently |
|  |  | Intentional rejection of childhood parenting models |
|  |  | Recognizing risk and choosing self-control |
|  | The Enduring Emotional Consequences of Childhood Violence | Navigating bitterness and bond |
|  |  | Emotional coldness and distance |
|  | Navigating Childhood Violence Through Suppression and Alertness | Mental violence and domination |
|  |  | Hypervigilance and emotional insecurity in the family |
|  |  | Childhood awareness of injustice |
|  |  | Self-perception and emotional pain in childhood |
|  |  | Silencing of childhood trauma |
|  |  | Coerced emotional suppression in childhood |
|  | Cultural, Familial, and Social Transmission | Structural vulnerabilities and violence in childhood |
|  |  | Violence as discipline in history |
|  |  | Violence between siblings in childhood home |
|  |  | Cultural inheritance of violence |
|  | Creating the Family I Deserved: Intentional Healing Through Parenthood | Self-healing through emotionally attuned caregiving |
|  |  | Safe, supportive, and non-violent partner |
|  |  | Emotionally attuned parenting |
|  |  | Non-violent parenting |
|  | Education and Support Systems in Healing and Empowerment | Education as professional identity |
|  |  | Support systems as a foundation for change |
|  |  | Healing through therapy and self-reflection |
|  |  | Healing through social functioning |

**Supplementary table 3.** Main themes and subthemes behind pathway 3

| **Pathway 3** | **Main themes** | **Subthemes** |
| --- | --- | --- |
|  | Struggling with Control and Emotional Regulation | Remorse and emotional accountability |
|  |  | Justifying and minimizing violence |
|  |  | When pressure breaks control |
|  | The Legacy of Violence | Emotional maltreatment in current family |
|  |  | Physical maltreatment in current family |
|  |  | Recognizing the cycle of violence |
|  |  | Emotional detachment and boundary-setting in parental relationships |
|  |  | Isolation and mistrust in relationships |
|  | Childhood Foundations of Trauma and Emotional Suppression | Suppressing emotions in childhood |
|  |  | Emotional pain and insecurity in childhood |
|  |  | Physical maltreatment in childhood |
|  |  | Living with the darkness |
|  | Breaking the Cycle | Learning to manage anger and non-violent coping strategies |
|  |  | Turning points toward change |
